# Building digital twins of the human immune system: toward a roadmap

**DOI:** 10.1038/s41746-022-00610-z

**Published:** 2022-05-20

**Authors:** R. Laubenbacher, A. Niarakis, T. Helikar, G. An, B. Shapiro, R. S. Malik-Sheriff, T. J. Sego, A. Knapp, P. Macklin, J. A. Glazier

**Affiliations:** 1 Department of Medicine, University of Florida, Gainesville, FL USA; 2 Université Paris-Saclay, Laboratoire Européen de Recherche pour la Polyarthrite rhumatoïde - Genhotel, Univ Evry, Evry, France; 3 Lifeware Group, Inria, Saclay-île de France, 91120 Palaiseau, France; 4 Department of Biochemistry, University of Nebraska-Lincoln, Lincoln, NE USA; 5 Department of Surgery, Larner College of Medicine, University of Vermont, Burlington, VT USA; 6 European Bioinformatics Institute, European Molecular Biology Laboratory (EMBL-EBI), Hinxton, Cambridge, UK; 7 Biocomplexity Institute and Department of Intelligent Systems Engineering, Indiana University, Bloomington, IN USA

**Keywords:** Translational research, Computational models

## Abstract

Digital twins, customized simulation models pioneered in industry, are beginning to be deployed in medicine and healthcare, with some major successes, for instance in cardiovascular diagnostics and in insulin pump control. Personalized computational models are also assisting in applications ranging from drug development to treatment optimization. More advanced medical digital twins will be essential to making precision medicine a reality. Because the immune system plays an important role in such a wide range of diseases and health conditions, from fighting pathogens to autoimmune disorders, digital twins of the immune system will have an especially high impact. However, their development presents major challenges, stemming from the inherent complexity of the immune system and the difficulty of measuring many aspects of a patient’s immune state in vivo. This perspective outlines a roadmap for meeting these challenges and building a prototype of an immune digital twin. It is structured as a four-stage process that proceeds from a specification of a concrete use case to model constructions, personalization, and continued improvement.

## Introduction

Today, nearly every industry that deals with complex technologies use sophisticated “digital twin” computer simulations to forecast how individual pieces of equipment should perform under ever-changing real-world conditions. For instance, a digital twin of a jet engine receives continuously updated operational data streamed over the “internet of things” while the engine is in flight. Any deviation between the digital twin’s prediction and the actual engine’s state can provide an early warning of a potential problem, which can be resolved before it becomes critical/catastrophic.

The medical analog is only in its infancy. Medical digital twins could find many uses and take many forms. They could predict disease trajectories in individual patients, allowing diagnosis before the onset of serious symptoms. They could be used to optimize the timing of suggested medical care and to investigate the effects of potential treatments in a patient-tailored manner. They could help identify biomarkers or elucidate drug mechanisms of action. They could be data-driven or based on mechanistic computational models of biological function, or a combination of both. Currently, digital twins of the human heart improve diagnosis, prognosis, and therapies^1,2^. Automated workflows for generating cardiac digital twins could serve as a blueprint for the generation of other types of medical digital twins. Another example of an operational medical digital twin is the artificial pancreas that aids Type I diabetic patients in insulin management^3^. Of particular importance are digital twins that capture key features of the immune system, with its ubiquitous influence on human disease. Being able to predict an individual’s immune response to infection or injury could be lifesaving in many ways, for instance in designing optimal individualized treatments^4^.

**Fig. 1:**
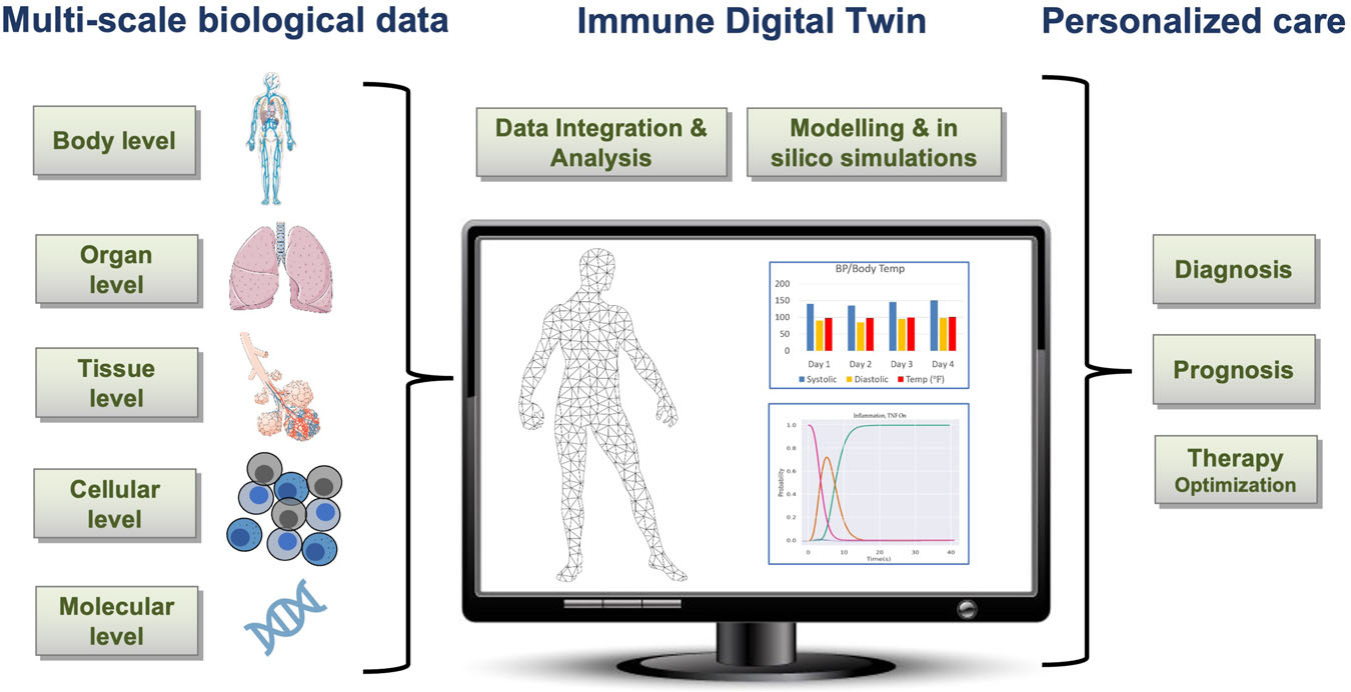
The construction and use of a digital twin. At each relevant physiological scale, known biology and relevant mechanisms are characterized through data collection that informs one or more computational models. The models at the individual scales are then integrated into a comprehensive multiscale base model. In the second step, this base model is personalized by parameterizing it with data collected from an individual patient. The resulting digital twin can then be used for clinical decision-making for this patient. (The images are public domain images from Servier Medical Art (https://smart.servier.com/smart_image/tendon-anatomy/), https://all-free-download.com/free-vector/flat-screen-computer-monitor.html, and https://pixabay.com/vectors/man-male-boy-human-people-persons-2099114/). All other images were produced by the authors).

This perspective describes a high-level outline of a roadmap for building an immune digital twin (IDT), depicted in Fig. 1. It advocates a partnership between the international not-for-profit scientific community, the commercial sector, and government funding agencies. Such an approach requires a collaborative infrastructure and broad agreement on the nature of the final product. For instance, the recently formed European-led DigiTwin consortium, which includes academic, clinical, and industrial partners from 32 countries, aims to create digital twins for every European citizen for a range of conditions^5^, and could become a model for some aspects of a global consortium for immune digital twins.

## Development stages

**Fig. 2:**
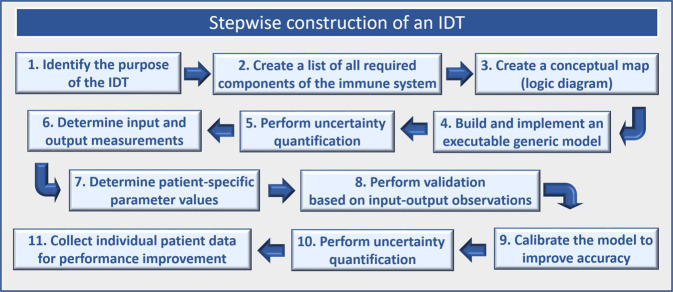
Immune digital twin workflow. The 11 steps in the four-stage workflow to construct an immune digital twin.

The construction of an immune digital twin will broadly follow the four-stage paradigm for industrial digital twin development (see Fig. 2).

**Stage 1:** Define a specific application and construct an appropriate generic template model.

The crucial first step is to determine a specific application of a personalized immune simulation model. Applications can be “online,” e.g., to predict the efficacy of a particular treatment based on frequent measurements of a patient’s condition, or “offline,” e.g., to develop novel drugs using simulated patient populations. Defining the application helps to narrow down the components of the immune system to include in the model, and to define the data available for model construction and operation, resulting in a generic “specification” of the digital twin design.

As there are many patient-specific immune system measurements that are currently infeasible, the choice of application is crucial for success.

The design steps are as follows.

1. Identify a specific purpose for the IDT. For example, it could be used to identify how to generate an immune response sufficient for effective viral clearance while avoiding excessive inflammatory tissue damage in a given patient. Identify all required inputs and read-outs of the IDT for the intended purpose.
2. List all components of the immune system, organ systems, physiology, and any organismal features, as well as their processes (behaviors and interactions), required to capture the mechanisms and features relevant for the intended purpose and the available interventions.
3. Create a conceptual map of the generic model that integrates all these components and features.
4. Build and implement an executable version of the generic model, parametrize and validate it, using data from humans, if available, as well as animal models and other experimental systems.
5. Carry out uncertainty quantification of model behavior.
  **Stage 2:** Personalize the template model to an individual patient.
  The calibration and contextualization of the generic template model with appropriate patient-specific data lead to a patient-specific digital twin. This stage creates an IDT model prototype that replicates key relevant features of an individual patient and passes an initial validation. The steps are as follows.
6. Determine patient measurements needed as IDT inputs and their frequency. Determine model outputs and their frequency. Inputs could consist of one-time measurements, such as immune cell counts to determine immune status at the time of a particular therapeutic intervention, or frequently repeated measurements, such as blood cytokine levels. Outputs could consist of one-time binary outputs, such as TREAT/DO NOT TREAT, or dynamic outputs such as deviation over time from a predetermined set of health parameters.
7. Determine any patient-specific parameter values or ranges of values in the IDT, such as determinants of liver function, as well as anatomical or other characteristics that are part of the model, and integrate them.
8. Perform an initial validation of the model by collecting a sufficient number of input/output values to test IDT performance. Adjust model characteristics as needed.
  **Stage 3:** Final IDT testing and uncertainty quantification.
  This stage requires two steps.
9. Test the IDT extensively under a variety of conditions and adjust model features and parameters as needed to improve accuracy.
10. Perform uncertainty quantification of the model. There are likely to be sources of parametric, structural, algorithmic, and observational uncertainties in the IDT. It is crucial to carry out a well-designed program to reduce and estimate the effect of these uncertainties on model predictions.

**Stage 4:** Continue to collect individual patient data for ongoing improvement of the IDT.

Ongoing collection of additional patient data over time of the type used to calibrate and personalize the generic model provides a richer data set to further reduce model uncertainty. This stage also addresses the fact that each patient is changing over time, and their IDT needs to be recalibrated periodically. Thus, Stage 4 represents an ongoing learning process to improve model performance and allow the IDT to evolve with the patient.

This process creates an IDT prototype for use in the fashion specified for the purpose determined at the outset. But a medical digital twin will likely never be a finished product. One critical reason for the success of industrial digital twins is that they incorporate a mechanism for continuous model improvement, where systematic deviations between model predictions and actual observations are used to refine model parameters or to suggest improvements to model structure/hypotheses. As a result, each time any digital twin is used, all digital twins of the same type improve based on its experience in operation. Stage 4 captures this feature.

Because immune responses to different situations share many components and processes, the components of an IDT developed for one purpose may be reused to facilitate the construction of IDTs for other purposes. As the repertory of available components grows, one could construct a “core” model that represents commonly shared features of the immune response which can then be extended and customized with additional components for specific applications.

## Requirements and considerations

### Collaborative and federated model development and validation

The effort outlined here will require a large, carefully coordinated, collaborative effort between immunologists, modelers, clinicians, computational scientists, and software engineers. The required expertise and resources go beyond the capabilities of any single organization, whether commercial or academic. In many ways, establishing the team-science human infrastructure is the most important and most difficult project feature. A large part of the expertise and knowledge base for IDTs resides in the academic community. Academic laboratories around the world have independently developed computational models of aspects of the immune response in various contexts^6–14^. We need to support the integration of these “local” efforts by developing enabling infrastructure and connecting the modeling community closely with experimentalists and clinicians. Spurred on by the SARS-CoV-2 pandemic, several community efforts emerged to create large-scale computational models of within-host disease dynamics, with approaches ranging from discrete to continuous mathematical modeling, e.g., refs. ^15–23^. Much can be learned from these experiences.

The organizational structure must provide leadership in mapping out project tasks and managing execution while respecting the autonomy of individual collaborators. Contributors of models, data, and clinical work must receive appropriate credit for their efforts and retain intellectual ownership of their contributions while enabling their sharing, interconnection, and reuse. How these issues will be resolved will depend in large part on whether an organizational structure relies on a dedicated implementation team that leverages community expertize and extant models and data, or, instead, emphasizes enabling individual laboratories to themselves develop and integrate models, code, and data for an IDT project.

### Software infrastructure

Lowering barriers to collaboration requires the development of software infrastructure to encourage and support model integration. Such shared infrastructure should allow participants to develop individual component models which can interconnect with each other with minimal extra effort and overhead. Implementation of common standards is one way to accomplish this. This infrastructure could be grafted onto the conceptual map of the IDT and can provide a template for what component models need to be included where. Dependency between sub-models needs to be minimized to avoid problems during model simulation.

Creating this infrastructure requires the solution of many specific technical problems. For example, a pipeline-like approach could help create a unified space for launching simulations of many different model components^24–26^. A shared set of analytical tools might include topological analyses to delineate model structure and identify core modules and concepts, dynamic analyses of discrete logic-based models^27–30^, and systems of ordinary differential equations (ODEs) representing different biological phenomena (gene regulation, signaling, and metabolism) across time scales.

Several available technologies address some aspects of IDT infrastructure. The IDT template model could be composed of many different modules combined in a plug-and-play fashion, for instance using the Python-based hub-and-spokes architecture proposed in ref. ^31^. This approach has the advantage that all sub-models access and modify the global model state only, without interacting with each other directly. Cell Collective^32^ is a web-based modeling platform for collaborative construction, simulation, and analysis of large-scale dynamic models of biological processes which supports logical and constraint-based models and extensive model annotation at multiple levels, enabling users to provide detailed biological evidence that supports model interactions, in addition to other logical model resources^33^.

### Modeling considerations

IDTs are complex computational structures whose sub-components may include both mechanistic models and/or data-driven statistical and machine learning algorithms. The specific use and data availability for each component will determine how it is implemented. For instance, well-developed AI approaches to image/pattern analysis could interpret CT or MRI images of infectious lesions, while mechanistic physiological and pharmacokinetic models would be appropriate to describe drug absorption distribution metabolism and elimination or signaling and feedback between immune cells components. Other cases may employ a hybrid approach that incorporates as many known mechanisms as possible, with data-driven models standing in for missing mechanistic information.

### Information management

Effective and comprehensive information management is essential if a project of this complexity is to be used in life-critical biomedicine and healthcare delivery. One solution is to use a dedicated team that manages information intake, organization, curation, and access by contributors. A more open approach would provide an information management system that allows all contributors to upload and curate their own information, linking it to other parts of the project through navigation tools. Both have their own advantages and disadvantages to be weighed in making a choice. Perhaps the most important component will be a flexible specialized repository of models and data sets, similar to existing general repositories such as Cell Collective^32^, SimTk^34^, and BioModels^35^. The European Bioinformatics Institute (EMBL-EBI) is developing a “search engine” for computational models, ModeleXchange, which will be able to integrate and search across model repositories to find models of interest.

## Discussion

This perspective provides a high-level roadmap for developing immune digital twin technology for biomedical and clinical applications. While IDTs present formidable scientific and technical challenges, even rudimentary instantiations for a given application would help focus ongoing data collection and other improvements, leading to progressively better-personalized simulation models over time. We propose the establishment of a *Consortium for Predictive Immunology* that works to integrate the currently fragmented trans-disciplinary researchers to make IDTs a reality. This collaborative human infrastructure, closely integrating modeling and clinical deployment would help transform the nature of biomedical research, greatly accelerating the bench-to-bedside pipeline and enabling currently unreachable medical goals. It would also provide numerous opportunities for new training paradigms for both biomedical and computational researchers.

We envision a two-stage project extending over 7 years. An initial planning effort will extend over 2 years. During this time the application(s) will be determined, a conceptual map of the IDT will be established, the necessary infrastructure will be put in place, and the composition of the group of collaborators will be determined. The first step is to assemble key stakeholders to define goals and approaches: modelers, immunologists, clinicians, software engineers, commercial entities, and funders. Our aim is to build: (1) a small number of promising applications of IDT technology; (2) a steering group that can initiate and coordinate the next steps; and (3) an outline of available funding sources, beginning with funds for a planning phase. During the next 3 years, the Consortium will construct and validate a prototype version of the IDTs and their supporting computational infrastructure. The final 2 years will validate the IDTs in patient trials. The Consortium needs to be funded as a coherent single project with distributed performance sites, including resources for modeling and software development, as well as experimental and clinical testing and validation.

We emphasize that the challenges to be met in such a project are formidable. Its cost and complexity are comparable to the Cancer Moonshot Program, funded at $1.8 B US Dollars through the US National Institutes of Health for a duration of 7 years. Depending on initial applications, it might require novel measurement technologies. And major new scientific discoveries and technological developments will be needed to achieve immune digital twins that are robust and accurate enough to approach industrial standards.

## Conclusion

Immune system digital twin technology is within our reach. Since the immune system plays a role in essentially all major diseases faced by humankind, including infectious, heart, respiratory and autoimmune diseases, the impact will potentially be extremely high. The time to begin is now.
